# Drain Structural Defect Detection and Mapping Using AI-Enabled Reconfigurable Robot Raptor and IoRT Framework

**DOI:** 10.3390/s21217287

**Published:** 2021-11-01

**Authors:** Povendhan Palanisamy, Rajesh Elara Mohan, Archana Semwal, Lee Ming Jun Melivin, Braulio Félix Gómez, Selvasundari Balakrishnan, Karthikeyan Elangovan, Balakrishnan Ramalingam, Dylan Ng Terntzer

**Affiliations:** 1 Engineering Product Development Pillar, Singapore University of Technology and Design (SUTD), Singapore 487372, Singapore; povendhan_palanisamy@mymail.sutd.edu.sg (P.P.); rajeshelara@sutd.edu.sg (R.E.M.); archana_semwal@sutd.edu.sg (A.S.); melvin_lee@sutd.edu.sg (L.M.J.M.); brauliofelixgomez@gmail.com (B.F.G.); selvasundari@layorz.com (S.B.); e_karthikeyan@sutd.edu.sg (K.E.); 2 LionsBot International Pte. Ltd., #03-02, 11 Changi South Street 3, Singapore 486122, Singapore; dylan@lionsbot.com

**Keywords:** reconfigurable robot, defect inspection, drain inspection, deep learning, computer vision, mapping

## Abstract

Human visual inspection of drains is laborious, time-consuming, and prone to accidents. This work presents an AI-enabled robot-assisted remote drain inspection and mapping framework using our in-house developed reconfigurable robot Raptor. The four-layer IoRT serves as a bridge between the users and the robots, through which seamless information sharing takes place. The Faster RCNN ResNet50, Faster RCNN ResNet101, and Faster RCNN Inception-ResNet-v2 deep learning frameworks were trained using a transfer learning scheme with six typical concrete defect classes and deployed in an IoRT framework remote defect detection task. The efficiency of the trained CNN algorithm and drain inspection robot Raptor was evaluated through various real-time drain inspection field trials using the SLAM technique. The experimental results indicate that robot’s maneuverability was stable, and its mapping and localization were also accurate in different drain types. Finally, for effective drain maintenance, the SLAM-based defect map was generated by fusing defect detection results in the lidar-SLAM map.

## 1. Introduction

Drains are the primary conduits transporting sewage, rainwater, and other liquid waste to the point of disposal in any urban environment. Hence, a routine drain inspection is a prerequisite in today’s scenario. Structural deterioration is a continuous process that reduces the load-bearing capacity of drains and gives rise to various kinds of defects, which further leads to potential infrastructural damage [1]. The traditional method for defect detection in open and closed drains and sewers is human visual inspection. However, it is a labor-intensive task, time-consuming, and difficult for humans to monitor many locations simultaneously over extended periods. Additionally, the workforce shortage is another issue that drain management companies face due to low wages, health issues, and being prone to high risk of accidents in a complex environment. In [2], the economic survey indicated that demand for remotely operated drain inspection devices is expected to grow at a compound annual growth rate (CAGR) of 8.3% from 2020 to 2027. The factors mentioned above are foreseen to drive the demand to automate drain inspection and defect mapping tasks.

In the literature, various algorithms and tools are reported to automate drain defect detection and mapping tasks. Closed-Circuit Television (CCTV) [3,4,5,6,7,8], sonar [9], laser scanner [10], infrared [11], and computer vision algorithms with robot-assisted remote inspection [12,13,14,15,16,17] are commonly used methods. Among them, computer vision-based robot-assisted remote inspection is a widely used method in the industry and has been classified into two categories: the traditional approach (non-learning) and the learning-based approach. Here, the traditional approach used feature descriptors such as Scale Invariant Feature Transform (SIFT) [18], Speeded Up Robust Features (SURF) [19], Features from Accelerated Segment Test (FAST) [20], Hough transforms [21], etc., for defect detection. On the other hand, learning-based approaches such as Machine Learning (ML) and Deep Learning (DL)-based frameworks are widely used in various defect inspection applications. Deep learning assists in achieving greater accuracy and often requires less expert analysis and fine-tuning [22,23].

Generally, CCTV and fixed morphology robot are widely used tools for computer vision-based robot-assisted drain inspection and defect mapping application [13,14,15,16,23,24]. However, deploying and maintaining CCTVs for long-range drain networks is a challenging task. More cameras are required to cover a more extensive drainage system area and to accurately pinpoint the exact location of the defect in the drain. On the other hand, fixed morphology robot-based drain inspection has some practical shortcomings. The fixed morphology robots are mostly designed for sewer pipe or tunnel inspection, which are hard to fit into uneven complex open and closed drain environments for inspection. As a result, during the inspection, versatility and efficiency is limited.

Reconfigurable robots are more flexible than fixed-size robots. In recent years, reconfigurable morphology-based robots have been widely used for various automation applications including cleaning indoor and outdoor environments [25,26], inspecting built environments [23,27], ship hull inspection, aircraft structural inspection [28], etc. From the literature, it is realized that designing a reconfigurable morphology-based robot is an optimal solution to overcoming the shortcomings mentioned earlier. Through reconfigurable robots, we can access complex drain structures and constricted drain environments by changing the morphology.

To the best of our knowledge, there is no direct case study using AI-powered reconfigurable robots for defect detection and mapping in a drain environment. This work presents an AI-enabled robot-assisted remote drain inspection and defect mapping using our in-house developed reconfigurable robot Raptor. This reconfigurable robot is specially designed for inspecting complex drain environments with the help of a DL algorithm and the SLAM technique and generates a SLAM-based defect map.

This paper is organized as follows: Section 1 presents the introduction and motivation to conduct this work. The literature review is presented in Section 2. Section 3 and Section 4 describes the methodology and overview of the proposed system. The experimental setup, findings, and discussion are covered in Section 5. Finally, Section 6 concludes this research work.

## 2. Literature Survey

Generally, a computer vision system is an key component in automating defect detection and mapping applications. This section describes a literature survey of computer vision-based defect detection and mapping schemes using traditional and learning-based approaches. In the traditional approach, various method were studied to identify defects for various application. In [29], Chinthaka et al. presented an image-based automatic road crack detection framework for achieving smooth driving on deformed roads. Otsu’s binarization, a discriminant analysis method, was employed for crack extraction from crack images of each sample. A detection rate of 94% was achieved. In another study [30], the authors presented a novel method to detect moving objects using a 360-degree view camera. Image processing steps such as gamma correction, background subtraction, noise removal, and confirmation by contour determination were used to detect the moving object. Moving objects almost within 10 m could be detected with a high detection rate. Wang et al. in another study presented an automatic detection of rail-surface cracks [31]. The Simple Linear Iterative Clustering (SLIC) algorithm was applied to generate superpixels of raw rail images to identify cracks. Five classification algorithms, the Support Vector Machines (SVM), Neural Networks (NN), Random Forests (RF), Logistic Regression (LR), and Boosted Tree (BT), were compared, in which RF provided the best performance. In traditional computer vision techniques, it is mandatory to choose the best features describing different classes of objects. As the number of classes to classify increases, feature extraction becomes more and more difficult.

The traditional computer vision system is a key module that can be combined with artificial intelligence using Machine Learning (ML) and a Deep Learning (DL)-based framework for different inspection applications. A DL technique is proposed for the automatic detection of multiple sewer pipe defects in [4]. The authors used a Faster Region-based Convolutional Neural Network (FR-CNN). The mean average precision increased to 83% by increasing the dataset size and convolutional layers and by adjusting the hyperparameters. Likewise, a deep learning-based framework, the YOLOv3 algorithm, was used to detect sewer pipe defects. The training dataset for the model contained six different defects in the drain, which were broken, holes, deposits, cracks, fractures, and roots, and the framework detected the defect with a mean Average Precision (mAP) of 85.37% [32]. Syed et al. suggested a deep learning framework for classifying defects (defect longitude, debris silty, joint faulty, joint open, lateral protruding, and surface damage) in underground sewer pipes. The CNN was fine-tuned before training and trained on a total of 47,072 images, which assisted in recording the highest accuracy of 96.33% [7].

## 3. Methodology

**Figure 1:**
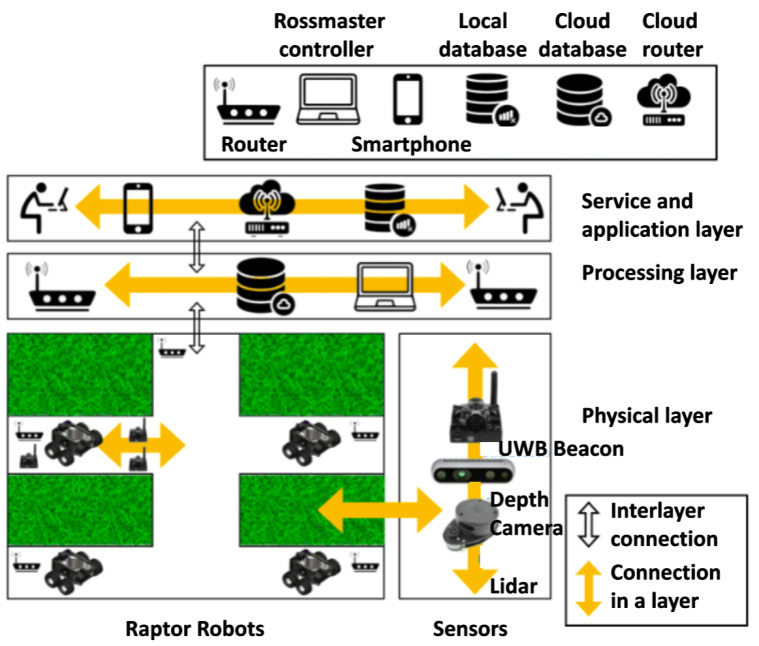
Overview of the drain inspection framework.

This work adopts the IoRT (Internet of Robotic Things)-based method for implementing a remote drain inspection and defect mapping. The IoRT is an advanced version of the Internet of Things (IoT). It is a combination of many technologies such as Cloud Computing, Artificial Intelligence (AI), IoT, and robotics technology [33]. A four-layer IoRT framework was used to set up the functional component of our proposed system. Figure 1 shows an overview of a four-layer IoRT-based drain inspection framework. It consists of (1) a physical layer, (2) a transmission layer, (3) a processing layer, and (4) an application layer. Here, the physical layer is represented by the robot and its sensors and actuators. Wireless communication between the robot and remote server was mapped in the transmission layer. The remote server is the processing layer where the DL-based object detection framework was employed to generate the drain defect map from Raptor-collected images along with the SLAM algorithm. Finally, the user interface is represented as the application layer.

## 4. Defect Detection and Mapping with IoRT Framework

### 4.1. Physical Layer

**Figure 2:**
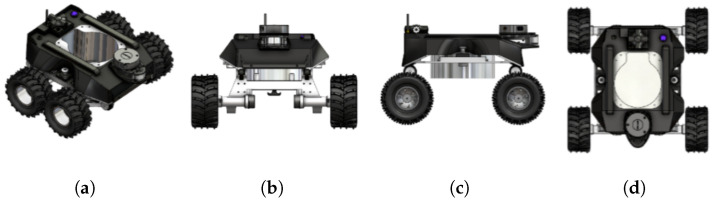
Different views of Raptor. (**a**) Isometric view; (**b**) Front view; (**c**) Side view; (**d**) Top view.

A fleet of autonomous mobile robots named ’Raptor’ was developed for visual drain inspection, capturing the drain’s wall and terrain and transmitting it to the defect detection algorithm. The robot ’Raptor’ facilitates the implementation of the physical layer in the proposed drain inspection system. Figure 2 shows the different views of Raptor. Typically, a drain inspection robot has many design constraints and challenges due to the complex drain infrastructure, uneven and slippery terrain, and heavy payload and should be able to perform complicated inspection tasks while being operated remotely. Furthermore, a vision sensor was attached with a pan-tilt mechanism to handle the narrow drain path. The pan-tilt mechanism mounted at a height allows for a broader view of the drain structures on either side. Table 1 shows the detailed specifications of the Raptor robot. The robot is lightweight (2.45 kg) and compact (390×350×200 mm) for easy maneuvering and offers maximum possible rigidity in inconsistent dimensions and terrain of the drain.

#### 4.1.1. System Architecture

**Figure 3:**
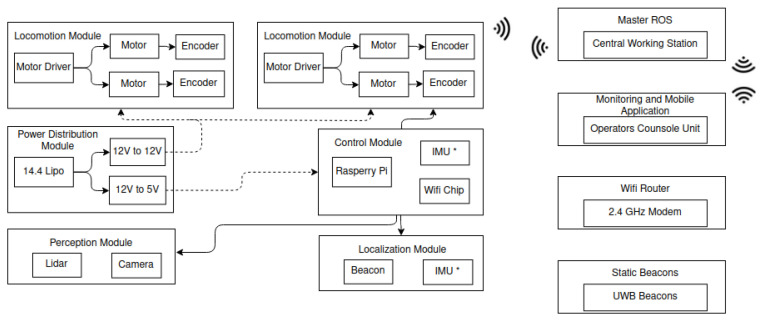
System architecture.

The system architecture of Raptor is depicted in Figure 3. It consists of different modules, including locomotion modules, the control unit, the power distribution module, the localization module, the collision detection and navigation module, and the reconfigurable module.

#### 4.1.2. Locomotion Module

The key design requirement of the locomotion module is to resist ground shocks and impulsive reaction loads. Therefore, all four wheels of the Raptor are powered by a DC metal gear motor 20 D ×46 L mm 12 V CB motor to prevent the locomotion module from struggling in the complex environment of drains. The motor’s power factor provides a maximum gradient of 20 to 25 degrees for continuous operation on uneven terrain. Furthermore, a fork with supporting ribs is mounted to hold the wheel motors and to distribute the load uniformly to the four wheels, and metal hubs with rims are employed to firmly secure the Raptor’s wheels. On the other hand, rubber tire tread is used to increase the amount of friction on the ground. The wheel’s diameter and the chassis height provide good ground clearance to overcome various obstacles on the path.

#### 4.1.3. Control Unit

The onboard control unit for Raptor was built using a Raspberry Pi single board computer and Robot Operating System (ROS) middleware. It processes the data from cameras, lidar, Inertial Measurement Unit (IMU), and Beacon modules and supports the robot in operating in telemetry and fully autonomous capabilities. While operating with the telemetry mode, the robot control system takes velocity and direction input commands from the operator via a remote server and outputs individual motor speed signals using the inverse kinematics method. On the other hand, while operating in autonomous mode, the remote server generates navigation trajectories via the NavFn global and DWA local planner. These trajectories in the form of waypoints are passed to the robot for autonomous navigation. Furthermore, the onboard control unit generates individual wheel speeds based on the trajectory and odometry information when operating in an autonomous mode.

#### 4.1.4. Power Distribution Module

The power distribution module comprises a four-cell Lithium-ion battery (14.4 V 2800 mAh) and two voltage regulators that generate 12 V for the motor driver and 5 V for the Raspberry Pi hardware.

#### 4.1.5. Localization Module

**Figure 4:**
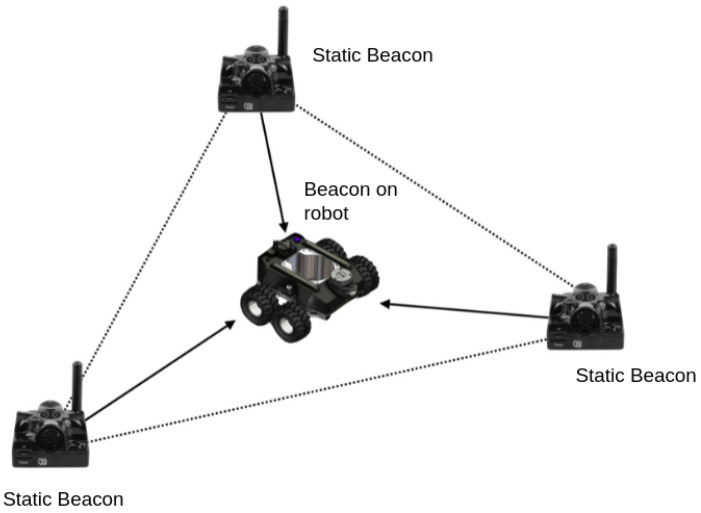
UWB localization.

Localization is an key function used to determine the robot position in a drain environment and used to compute the defect location. An indoor GPS is used to localize the robot position in a drain environment. Marvelmind’s UWB Super Beacons was adopted as the indoor GPS. It provides an accuracy of up to 2 cm. At least three stationary beacons are required to form a map, as shown in Figure 4. Here, one beacon should be fixed in the robot (mobile beacon), and the other beacons are positioned at different corners according to the drain structure. When the robot moves, the position of the mobile beacon changes in relation to the stationary beacons. These data are used to track mobile beacon positions. Along with the UWB beacon, absolute IMU, and wheel odometry of the robot, the data are fused together to localize the robot position accurately.

#### 4.1.6. Collision Detection and Navigation Module

The navigation stack of the autonomy layer uses a dedicated ROS node called move_base for path planning and obstacle avoidance. The move_base node links a global and local planner together to accomplish global navigation tasks. Move_base plugin implements an action that attempts to reach a geiven goal with a mobile platform. Running the move_base node on the Raspberry Pi results in a robot that attempts to achieve a goal pose within a user-specified tolerance with the robot base. The low-cost RP lidar A1 was mounted at the top of the robot to scan the surroundings, looking for any dynamic obstacles during its navigation. With lidar’s rotation frequency of 10 Hz and 8000 sampling points per second, the dynamic obstacles are accurately detected and are processed by the move_base algorithm to avoid them.

#### 4.1.7. Reconfigurable Module

**Figure 5:**
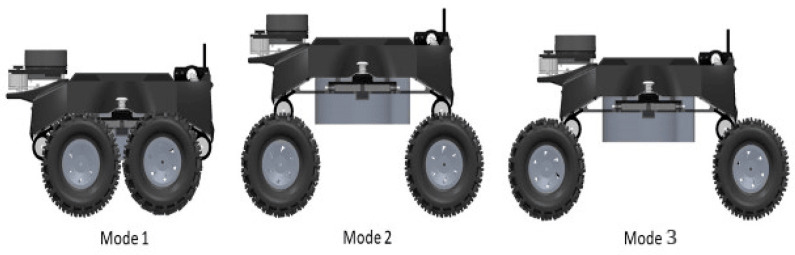
Three reconfiguration modes.

**Figure 6:**
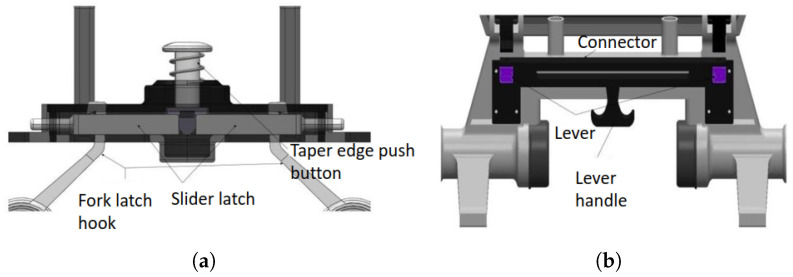
Reconfigurable mechanism. (**a**) Push button mechanism; (**b**) Lever mechanism.

The reconfiguration module plays a crucial role in Raptor functionality and is one of the vital contributions from this work. The manual reconfigurable module uses different stages based on the drain situation, as shown in Figure 5. Mode 1 is fully retracted, used for more stringent and narrow passages. Mode 2 is hybrid-open and used for areas that require maximum ground clearance. Finally, Mode 3, fully expanded, is used for more excellent stability. The robot has two mechanisms, which are push-button and lever mechanisms, as shown in Figure 6. As the user releases the push button, the latch slides horizontally and removes the fork latch hook. Once unlocked, the lever handle can be adjusted to the wheel’s position. This variation and flexibility due to the reconfiguration modes facilitate smoother maneuverability in different drain terrains. The design considersation of individual drain types and terrains with a simplistic approach for robots and the reconfigurable mechanism is an innovative and essential contribution compared with state-of-the-art robot research.

#### 4.1.8. Vision System with Pan-Tilt Mechanism

**Figure 7:**
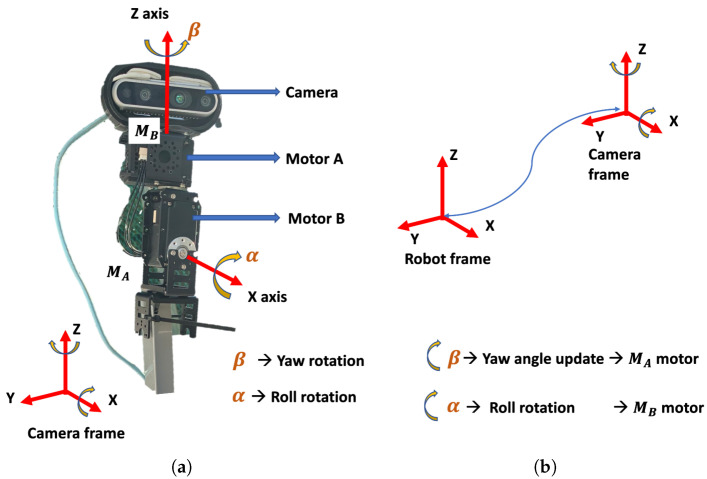
Pan-tilt mechanism. (**a**) Pan tilt camera setup; (**b**) Frames of reference transformation.

The robot vision system was built using a real-sense stereo camera fitted on top of the pan-tilt mechanism. Here, the pan-tilt mechanism was designed considering the broader field of view requirements in a drain environment with enabled high-precision angle control capabilities in two degrees of freedom. The pan-tilt mechanism consists of two servo motors (dynamixel mx-64), as shown in Figure 7. Here, motor A controls the degree of freedom (d.o.f) around the *z*-axis and motor B controls the d.o.f around the *x*-axis of the camera frame. These servo motors control the yaw and roll angles of the pan-tilt mechanism. The yaw rotation is represented by B and the roll rotation by alpha, as depicted in Figure 7a. The transformation between the camera frame of reference and the robot’s frame of reference was performed using the Tf transformation function supported by the ROS architecture. The offset information between the *x*, *y*, and *z*-axes and the pitch angle was static and provided at the beginning of the experiment. In contrast, the yaw and the roll angle changes dynamically due to the working of the pan-tilt mechanism. Adaptive transformation is a technique that was proposed to tackle this problem of continuous change in the coordinate frame transformation between the sensors and robot reference frames through continuous estimation and update of the transformation frames. The orientation of each motor was estimated through a 12-bit absolute encoder, which was used to calculate the β and the α of the camera mounted on motor A. These roll and yaw angles were used to update the tf between the camera frame and the robot coordinate frame. Hence, through precious encoders and an adaptive transformation technique, all of the defects detected by the camera were transformed and added to the map in the robot’s frame (Equation (1)).

(1) Drobotframe=Dcamera+T

(2) $$T=\left[\begin{array}{cccc} & R & & t\\ 0 & 0 & 0 & 1 \end{array}\right]=\left[\begin{array}{cccc} r_{11} & r_{12} & r_{13} & t_{1}\\ r_{21} & r_{22} & r_{23} & t_{2}\\ r_{31} & r_{32} & r_{33} & t_{3}\\ 0 & 0 & 0 & 1 \end{array}\right]$$

**R** and **T** in Equation (2) are the rotation and translation matrices of the transformation function.

### 4.2. Network Layer

The network layer links the physical layer (robot nodes), processing, and application layers; 2.4 GHz WiFi communication was used for all local communication. The D-Link 4G /LTE mobile router built-in WiFi router was used to establish the internet communication. It uses a USIM card to connect to the internet.

### 4.3. Processing Layer

The processing layer was built with a powerful processing unit (remote server or cloud server) to manage physical, transmission, and application layer requests. This layer handles the following tasks: ROS master function execution, defect detection, and fusion of the defect location on the lidar generated drain map.

#### Deep Learning-Based Defect Detection

**Figure 8:**
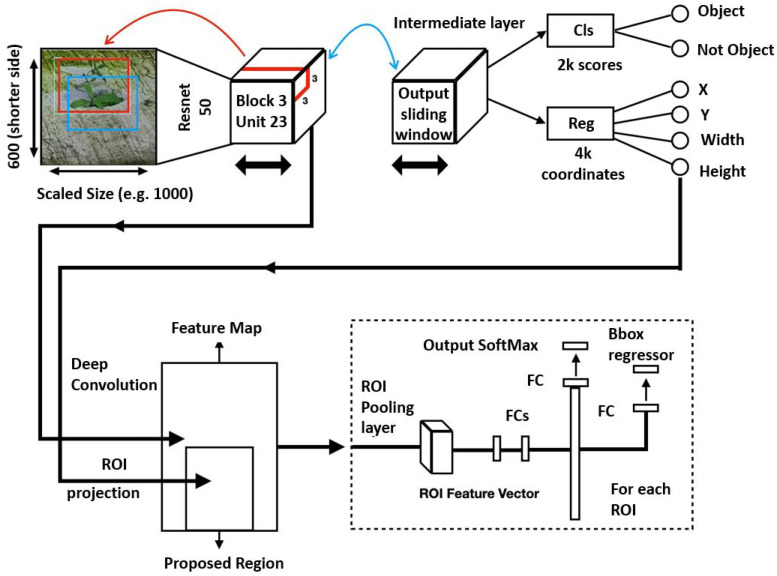
Functional block diagram of the Faster RCNN framework.

The Faster RCNN object detection algorithm (Figure 8) was used for detecting structural defects in the drain networks. Its two-stage object detector comprises three key components: feature extractor, Region Proposal Network (RPN), and detector head.

**Figure 9:**
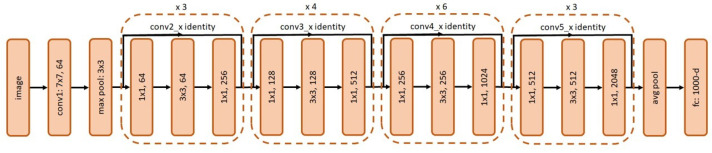
Network architecture of ResNet50.

**Figure 10:**
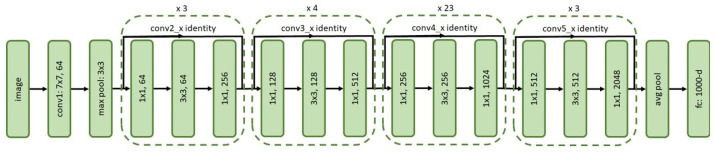
Network architecture of ResNet101.

**Figure 11:**
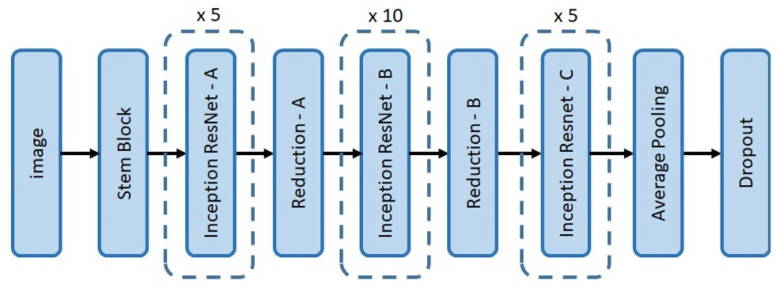
Network architecture of Inception-ResNet-v2.

Feature extractors: In our case study, the ResNet50, ResNet101, and Inception-ResNet-v2 DCNN frameworks (Figure 9, Figure 10 and Figure 11, respectively) were tested with Faster RCNN as the feature extractor. These feature extractors are pre-trained on the COCO dataset and were adopted from the TensorFlow model zoo [34].

**Figure 12:**
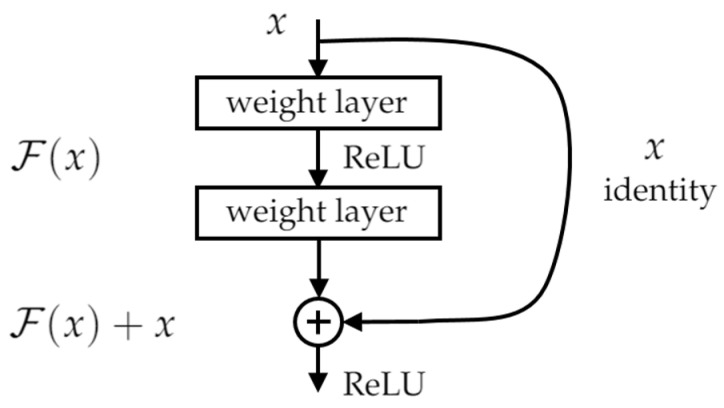
Residual learning unit.

All three frameworks utilize the residual learning unit, as seen in Figure 12, to alleviate the degradation of deep neural networks, with the main merit of this residual learning unit being the better classification accuracy without increasing the complexity of the model. He et al. [35] defined the residual learning unit as Equations (3) and (4), wherein the input and output dimensions of Equation (3) must be the same if the dimensions are different and the addition of $W_{s}$ allows for a linear projection to match the input and output dimensions. The only added complexity in the case of the residual learning unit is the negligible element-wise addition. The major difference between ResNet50 and ResNet101 is the different depths. With the introduction of a residual learning unit, Ref. [36] formulated Inception-ResNet-V2 based on the combination of their prior work with the inception and He et al. [35] introduced the residual connection. In the Inception-ResNet block, multiple-sized convolutional filters were combined via residual connections. The usage of residual connections avoids the degradation problem caused by deep structures and reduces the training time.

(3) $$y=F(x,\left\{W_{i}\right\})+x$$

(4) $$y=F\left(x,\left\{W_{i}\right\}\right)+W_{s}x$$

Region Proposal Network (RPN):

The RPN is a Fully Convolutional (FC) network trained to output object proposals with objectness scores. RPN uses a feature map from the feature extractor module to slide a small network of n×n spatial windows over the convolution feature map to predict the proposal regions. This operation reduces the feature map to a lower-dimensional feature. The lower dimension layer is connected to two sibling 1×1 fully connected layers, namely the classification layer (cls) and the regression layer (reg). The classification layer predicts output probability scores for two outputs (objectness and non-objectness), whereas the regression layer predicts the probability of four outputs (width, height, x-coordinate, and y-coordinate). The anchor box approach implements a pyramid of reference boxes in regression functions to address the issue of multiple scales and sizes. The anchor box scheme predicts multiple region proposals at each sliding window location anchored to the center location. Thus, the anchor box scheme generates a total of W×H×k anchor boxes. At each location of the sampling window, anchor boxes are trained to form around the present objects. For our use case, the k value is capped at nine. Furthermore, the Non-Maximum Suppression (NMS) algorithm is applied to filter out the overlapping bounding boxes.

Detector and Classifier Head: The final component of Faster RCNN consists of the Fast RCNN module, the Region of Interest (ROI) pooling layer, and a fully connected layer. The ROI pooling layer takes inputs from the shared convolution layer of the feature extractor module and region proposals from the RPN module. ROI extracts a fixed-sized feature map, generally 600 pixels on the shorter side. This feature map is used to detect and classify the bounding boxes in the following fully connected layer. Finally, object localization accurately eliminates non-desired bounding boxes.

### 4.4. Application Layer

The application layer is the user interface layer. It is used to control the robot from the remote location and to monitor the drain defect detection framework’s results. The Operator’s Console Unit (OCU) is the main graphical interface used to operate the system in the application layer, built using Unity 2019.4, a cross-platform engine for developing graphic programs. The OCU must be run on a workstation connected to the same network as the ROS master.

## 5. Experimental Setup and Results

This section elaborates on the experimental methods and results. The experiments were performed in five phases: dataset preparation, validation of the Raptor robot’s performance in a drain environment, training the drain inspection framework, evaluation of the trained framework in an offline and real-time field test in remote and AWS cloud servers, and comparison of the trained model with other object detection frameworks.

### 5.1. Dataset Preparation and Training

The training dataset comprises six different defects classes: potholes, cracks, intrusion cracks (plants in the crack and tree roots), honeycomb defects, and deposits. A total of 1800 images were collected for each class from various online sources and a real environment, where 1500 images were used in training for each class. Furthermore, the data augmentation process was applied to the collected dataset to mitigate the over-fitting issue and to enhance the learning rate of the detection framework.

#### 5.1.1. Training Hardware and Software Details

The drain inspection algorithm was developed using the open-source TensorFlow 1.15 machine learning framework, which runs on the Ubuntu 18.04.5 LTS operating system. The model was trained in a colab environment and used the following hardware for training: Intel (R) Xeon (R) CPUs at 2.00 GHz, 12.69 GB Random Access Memory (RAM), and an Nvidia Tesla P100 server graphics card (3584 CUDA cores).

#### 5.1.2. Parameter Configuration

The drain inspection model was trained using the transfer learning method and uses early stopping conditions. The transfer learning scheme uses pre-trained models directly as feature extraction preprocessing and integrates easily into the object detection framework. Furthermore, it decreases the training time for a neural network model and results in lower generalization errors. The feature extractor models such as ResNet50, ResNet101, and Inception-ResNet-v2 were pre-trained on the COCO dataset, and the layers from which the features are extracted can be seen in Table 2.

(5) D={X,P(X)}

(6) T={Y,P(y|x)}

In [37], the author defined a framework for understanding transfer learning using domain (Equation (5)), task (Equation (6)), and marginal probabilities. Given the source domain D_S_, the features learnt from the COCO dataset, and a source learning task T_S_, 80 different object categories were detected. The target domain of D_T_, features learnt from our drain defect dataset, and a target learning task T_T_ detected six different crack defects. Transfer learning can be used to improve the learning of new drain defect features. The various layers in which the features learned from D_S_ were extracted and fed into the Faster RCNN two-stage object detection framework, as shown in Table 2.

Under early stopping conditions, the models ResNet50, ResNet101, and Inception-ResNet-v2 were converged at 115532, 118356, and 276438 epochs, respectively, and the following training parameters were used: a batch size of 16, the RMS Prop algorithm with 0.9 momentum, and an initial learning rate of 0.004. The k-fold cross-validation procedure was used to evaluate the picture dataset. This approach divides images into k groups, with k-1 being utilized for training the network. The remaining one set is used for evaluating the model.

After training, the defect detection framework was tested in the IoRT framework-configured remote server, colab, and AWS cloud server. Here, the AWS G4dn instance was used to evaluate the IoRT framework in the cloud server experiment. It was powered by 8 NVIDIA T4 GPUs, 96 vCPUs, 100 Gbps networking, and 1.8 TB local NVMe-based SSD storage.

### 5.2. Offline Test

**Figure 13:**
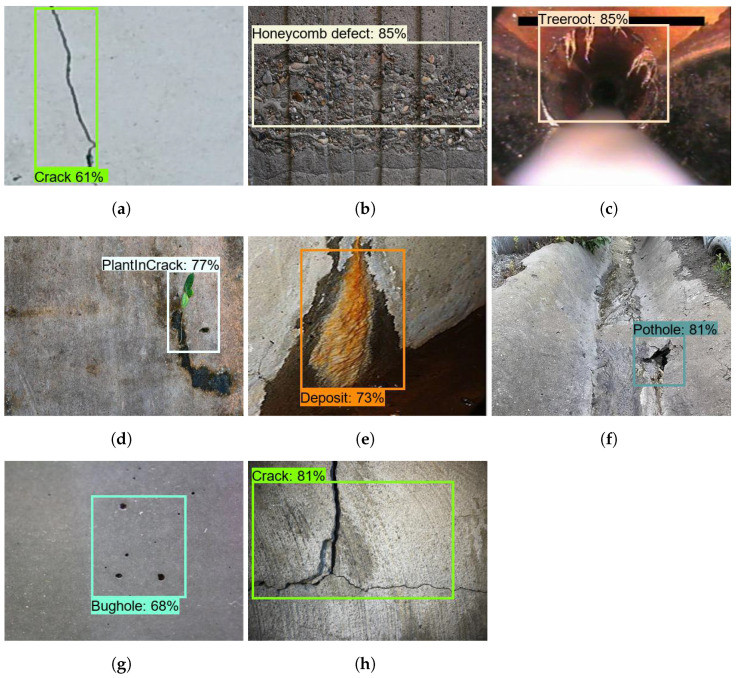
ResNet50 defect detection results. (**a**) Crack; (**b**) Honeycomb defect; (**c**) Tree root intrusion; (**d**) Plant intrusion; (**e**) Deposit; (**f**) Pothole; (**g**) Bughole; (**h**) Crack.

**Figure 14:**
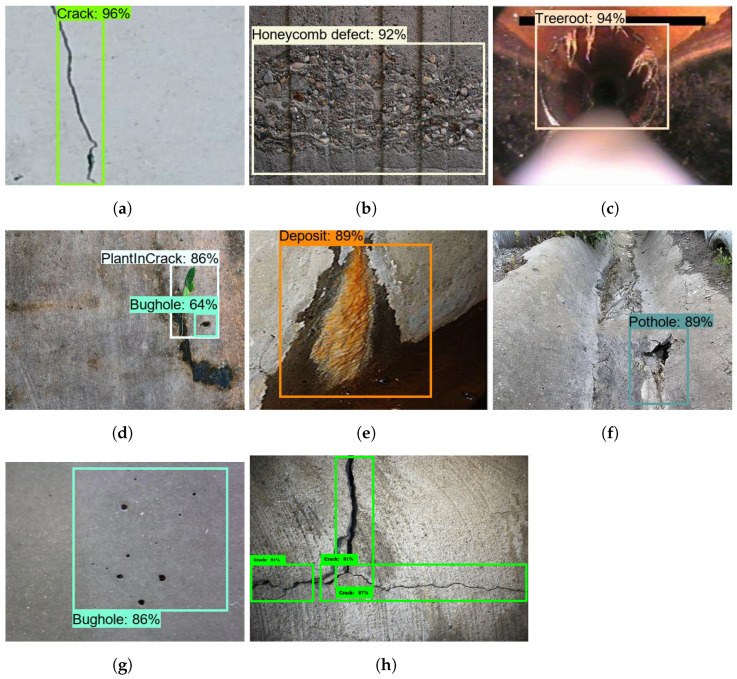
ResNet101 defect detection results. (**a**) Crack; (**b**) Honeycomb defect; (**c**) Tree root intrusion; (**d**) Plant intrusion; (**e**) Deposit; (**f**) Pothole; (**g**) Bughole; (**h**) Crack.

**Figure 15:**
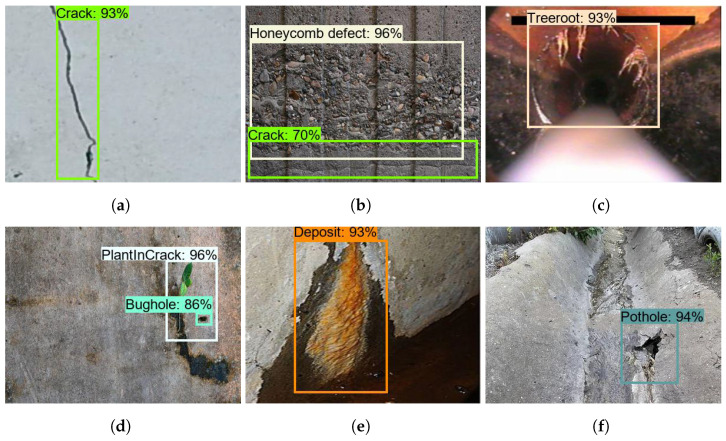

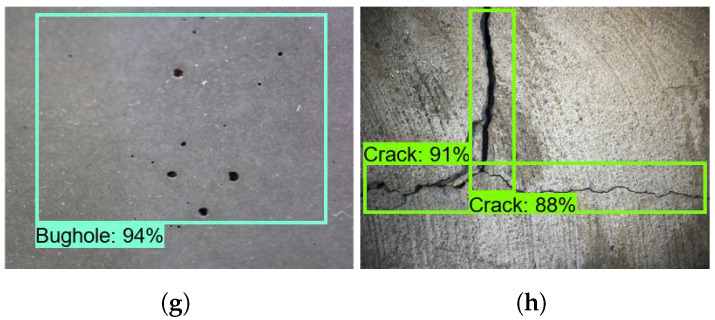
Inception-ResNet-v2 defect detection results. (**a**) Crack; (**b**) Honeycomb defect; (**c**) Tree root intrusion; (**d**) Plant intrusion; (**e**) Deposit; (**f**) Pothole; (**g**) Bughole; (**h**) Crack.

The offline test was performed using images containing defects that were collected from various web sources. There are 300 images used for each class to evaluate the model’s performance, and these images were not used to train the drain structural defect detection framework. Figure 13, Figure 14 and Figure 15 show the defect detection experimental results of three frameworks. Here, (a–h) indicate the concrete defect classes. Furthermore, the statistical evaluation results for the offline experiment are reported in Table 3. Here, accuracy, precision, recall, and $F_{measure}$ were used to evaluate the model [23,38].

The experimental results indicate that the three detection frameworks’ average detection accuracies are above 85%, that Faster RCNN ResNet50 scored 86.89%, that Faster RCNN ResNet101 scored 89.80%, and that Faster RCNN Inception-ResNet-v2 scored 92.67%. From the above analysis, we observe that the Faster RCNN 50 detection performance was slightly lower than that for Faster RCNN 101 and Faster RCNN Inception-ResNet-v2. Its performance score was affected due to the failure to detect a hairline crack in the concrete and due to false classification of defects such as cracks and tree roots. On the other hand, the detection accuracy of Faster RCNN Inception-ResNet-v2 was the highest. However, the Faster R-CNN Inception ResNet V2 inference time was relatively low when compared with the Faster RCNN ResNet101, as shown in Table 4 because the ResNet101 model has a dense feature extractor layer (101 convolutional layers) and uses 62.4 M parameters to infer the images. On the other hand, Faster RCNN Inception-ResNet-v2 uses 59.4 M parameters that use a combination of Inception and ResNet to extract features. In the final analysis, the offline experimental study shows that Faster RCNN ResNet inception v2 has a good trade-off between detection accuracy and computation time and is more suitable for drain defect inspection tasks.

### 5.3. Real-Time Field Trial

The real-time field trial experiments include a robot maneuverability test on different drains, evaluating the localization and mapping capabilities with multiple mapping algorithms and validating the trained model’s defect detection efficiency from live streaming videos.

#### 5.3.1. Maneuverability Test

**Figure 16:**
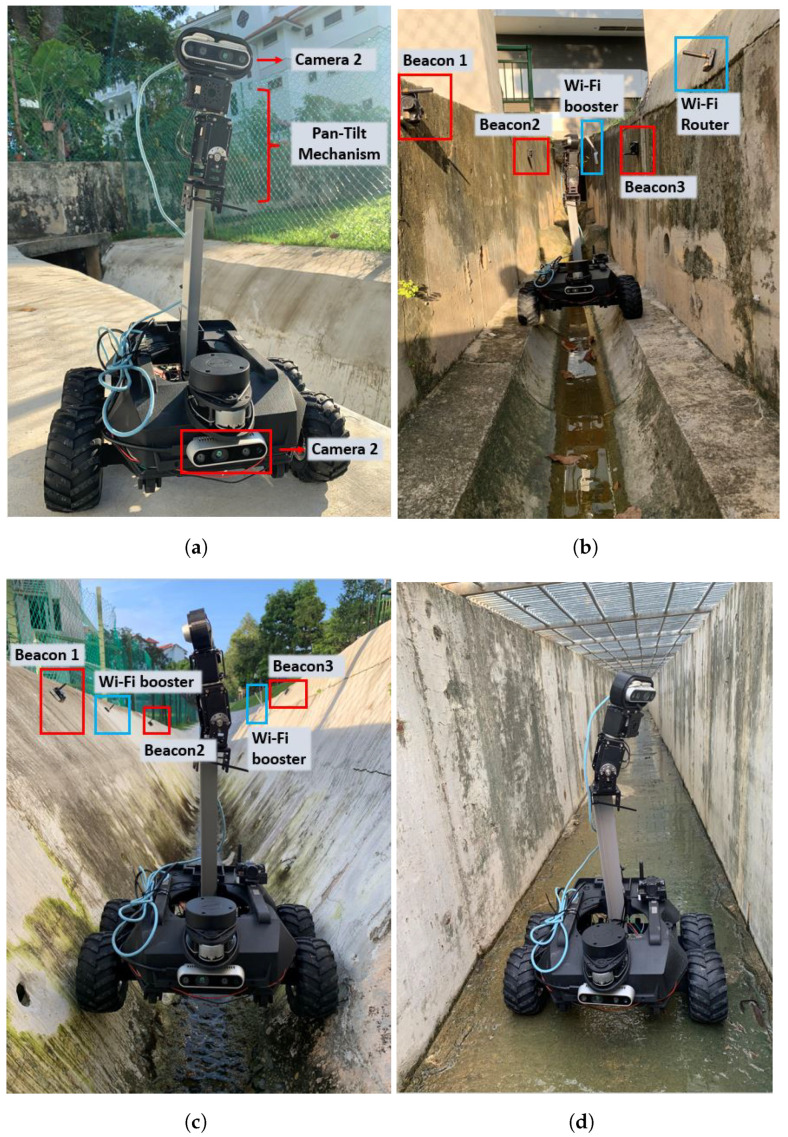
Raptor robot field experiments and defect detection in different drain types. (**a**) Robot and Pan-tilt mechanism; (**b**) Type S2 drain; (**c**) Type C7 drain; (**d**) Flat drain.

Robot maneuverability tests were conducted in three different drains types. Figure 16a shows the Raptor robot with a camera pan-tilt mechanism in the maneuverability test. The drain environment was prepared with beacon triangulation to improve the accuracy of localization and in-range WiFi signals to improve WiFi communication. Three static beacons and one local WiFi router were fixed on each straight stretch of the drain to avoid signal loss. According to the manufacturer, each mobile beacon must be in a range of at least 25 m from a stationary beacon for proper signal strength. Taking this into account, stationary beacons were placed on alternating sides of the drain every 20 m, which is shown in Figure 16b,c.

Figure 16b shows the robot tested in a cut-off type drain, also referred to as the C7 drain. Figure 16c presents the testing of the Raptor robot in an S2 type drain, which is V-shaped with a smooth bottom. Finally, Figure 16d shows the operation in a flat open drain. These drains are pathways for excess water collection, primarily from rainwater sources, with varying depths from 1 foot to 3 feet. The drain contains different terrain surfaces, such as sand, concrete, and wet and dry conditions. The tunnels in some of the drains are long and closed with sharp turns, slopes, narrow pathways, and level changes. All of the chosen drains are located inside SUTD University premises in Singapore, and 20 h extensive field tests were conducted, including all three types of drains. The experiments were randomly distributed in terms of drain types and a particular stretch of drain to avoid bias. Through these trails, it is observed that the Raptor robot is able to successfully traverse in all of the chosen terrain conditions and drain tunnel structures.

#### 5.3.2. Drain Mapping Algorithm Evaluation

A mapping algorithm evaluation was performed to identify the optimal algorithm for drain mapping and defect localization. The Hector SLAM (odometry-independent) and G mapping SLAM (odometry-dependent) algorithms are trialed and compared for map building efficiency. In autonomous navigation, mapping works in two environment situations, namely known environments and unknown environments. When the robot encounters an unknown environment, the localization happens simultaneously with respect to the generated map. Through SLAM, the robot located itself on a map and generated a virtual map of the place simultaneously. On the other hand, for a known environment, i.e., once the area is mapped for the first time, a subsequent inspection of the same drain is performed using this saved static map. This reduces the computation requirements and increases the localization accuracy of the robot.

**Figure 17:**
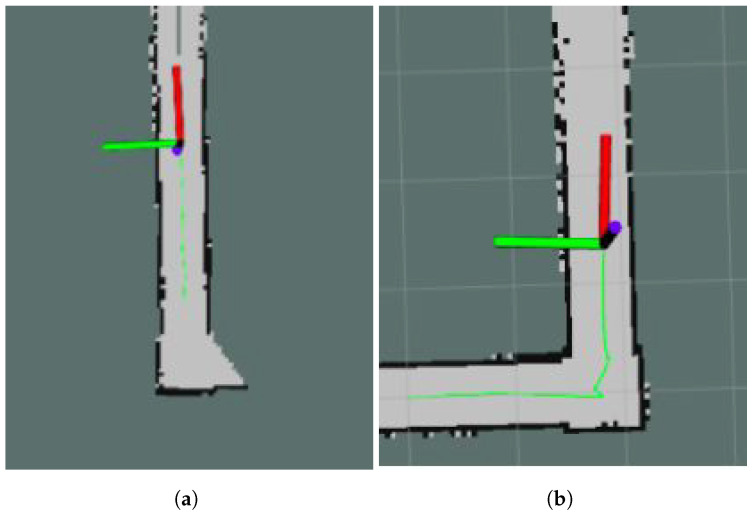
Robot ‘Raptor’ path tracking. (**a**) Pathplanning—right turn; (**b**) Pathplanning—straight.

Both algorithms (Hector SLAM and G mapping) were compared and tested for accurate loop closure in mapping and for smooth translation lines and sharp turns while localizing and path tracking. For the experiments, the robot was teleoperated in a controlled environment to form particular shapes, and the path was tracked using a green marker, as shown in Figure 17.

**Figure 18:**
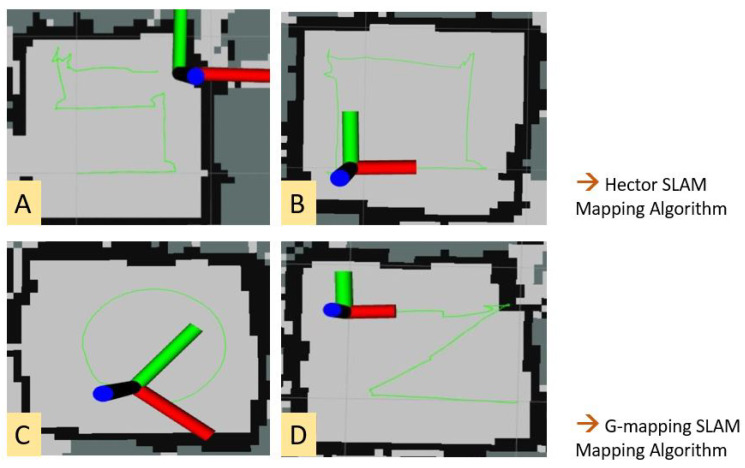
(**A**) S-Shaped; (**B**) Square; (**C**) Circle; (**D**) Z-shape.

Figure 18 shows the Hector and G mapping comparison results. Here, Figure 18A,B shows the Hector SLAM drain mapping results. It builds a grid map and localizes the robot using only the scan matching process as explained in [39]. Hector SLAM implements the Gauss–Newton approach for the scan matching algorithm, which finds the rigid body transformation of a laser scan’s endpoints. The optimal alignment is found by minimizing the transformation function (Equation (7)). The high update rate of the laser sensors combined with its accuracy and scan density play crucial roles for Hector SLAM.

(7) $$\xi^{*}=\underset{\xi }{arg min}\sum_{i=1}^{n}{\left[1-M\left(S_{i}\left(\xi \right)\right)\right]}^{2}$$

Figure 18C,D shows the G mapping [40] algorithm results. It uses Rao–Blackwellized particle filters to realize SLAM in mobile devices and robots. This approach computes the trajectory x jointly with the posterior p about the map. The respective function is defined as follows (Equations (8) and (9)).

(8) $$p(x_{1:t},m|z_{1:t},u_{1:t-1})$$

(9) $$x_{1:t}=x_{1},.......,x_{t}$$

where *z* is the observation from 1 to *t* seconds, obtained from the perception sensor, and the u1:t is the odometer measurement obtained from the position sensor of the mobile robot, Raptor. According to [41], this is summarized by the following factorization (10):

(10) $$p\left(x_{1:t},m|z_{1:t},u_{1:t-1}\right)=p\left(m|x_{1:t},z_{1:t},u_{1:t-1}\right)$$

This factorization is first used to estimate the trajectory of our Raptor robot and to compute the map using the trajectory information. Each of these factorized particles carries an individual map of the environment.

As can be observed, Figure 18A,B show more significant uncertainties in the path tracked, while Figure 18C,D shows consistency with the desired shape formation. Due to its inherent nature of position sensor, the Hector SLAM mapping shows unreliable results for the sensor combination used in the Raptor system. G mapping’s approach of proposal distribution is computed based on the robot’s recent observation, and robot movement improves the localization and path tracking accuracy. Based on these experiments, the most suitable SLAM approach for our sensor combination, G mapping, is selected and implemented. Localization happens via the G mapping algorithm through the sensor inputs from UWB beacons, the wheel encoders, and the IMU sensors.

Through the real-time drain field trails, the Raptor robot’s robustness and maneuverability were tested for operations in various drain structures. Furthermore, beacon localization, lidar mapping, and WiFi communication ensure uninterrupted operation in an unexplored drain environment.

### 5.4. Real-Time Defect Detection and Mapping

**Figure 19:**
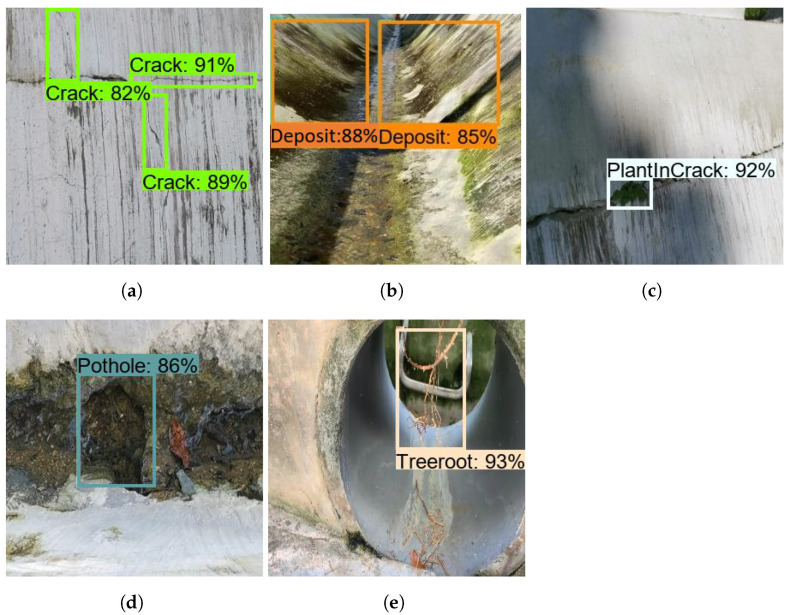
Real-time defect detection. (**a**) Crack; (**b**) Plant in crack; (**c**) Tree root; (**d**) Plant in crack; (**e**) Pothole.

**Figure 20:**
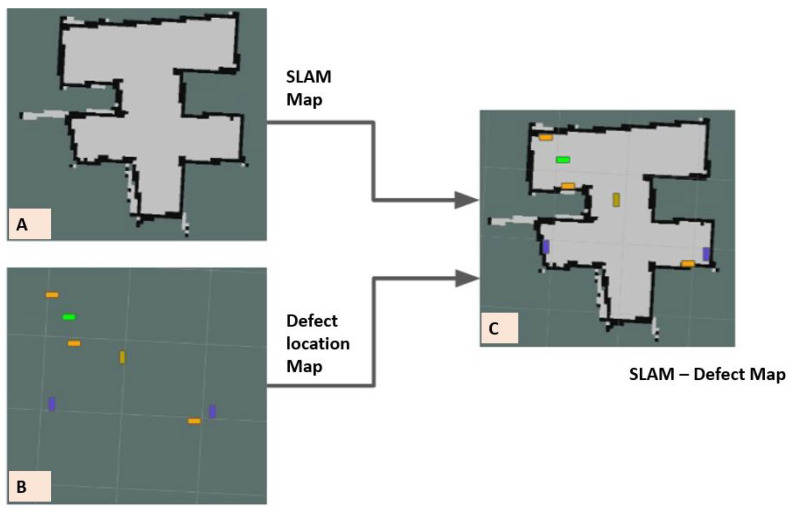
(**A**) SLAM Map; (**B**) Defect location Map; (**C**) SLAM-Defect Map.

The final stage of the field trial is to evaluate the drain inspection framework using Raptor-collected real-time drain environment video feed. The experiment was carried out in the three drain environments (closed drain, semi-closed drain, and open-drain), two lighting conditions (day and night), and two different climate days (after heavy rainfall and summer days). The robot was operated in semi-autonomous mode and controlled by a mobile APP connected via 4G internet communication through a remote server. The images captured by the robot are remotely analyzed by a drain inspection algorithm running on a high-powered GPU-enabled local server. Figure 19a–e shows the drain inspection algorithm field test results, where drains have a small pothole, crack, or intrusion cracks such as from plants or tree-root intrusion. The detection algorithm detected most of these defects from the Raptor robot-captured images with a good confidence level. The defect-detected image frame location coordinates are decoded from the header file and fused with the drain map. Figure 20 shows the defect fused on the drain map, where the color codes indicate the class of defects.

As in offline tests, Faster RCNN Inception-ResNet-v2 and Faster RCNN ResNet101 detect better than Faster RCNN ResNet50. The model detection accuracy for Faster RCNN ResNet50, Faster RCNN ResNet101, and Faster RCNN Inception-ResNet-v2 are 82%, 87%, and 89%, respectively. However, compared with offline tests, the model detection accuracy is relatively low due to the missed detection of defects.

For real-time tests, Table 5 shows the comparison of detection accuracy across various weather and lighting conditions. It was observed that the low light condition (night) induces higher false-positive rates and reduces true positive rates slightly under the same weather conditions. The same trend of higher false positives can also be observed independent of weather conditions. There is also a drop in true positive rates when comparing across the same light conditions but different weather conditions. In general, the higher miss detection is caused by object occlusion, blurring due to jerks in robot navigation when moving on an uneven surface, shadows, varying lighting conditions, and weather conditions.

#### Defect Mapping on SLAM Map

Figure 20 depicts the 2D SLAM defect map marked with various colors. Here, defects are marked as tiny squared boxes with six different colors. Two maps are used to generate the SLAM defect map, namely the SLAM grid map and the defect occupancy map, where the SLAM map is generated by the G mapping algorithm and x,y coordinates of a 2D map are used to create the defect location map.

Here, the grid-based SLAM map (Figure 20A) consists of three colored pixel regions, namely green, white, and black, representing unknown, unoccupied, and occupied (with obstacles) regions, respectively. The SLAM map is generated in the robot’s frame of reference, and the values of each grid in the SLAM map are given as follows.

(11) $$m^{\left(k\right)}=\left\{\begin{array}{cc} -1, & \mathrm{unexplored},\\ 0, & \mathrm{unoccupied},\\ 100 & \mathrm{occupied} \end{array}\right.$$

For generating the defect map, the distance calculated from lidar is equated to the identified defects and marked (Figure 20B) with respect to the corresponding angle/orientation on the map. Furthermore, different defects (as listed in Table 3), suggested by the machine learning algorithm, were labeled with the respective colors associated with them to facilitate easier identification and inspection. The defect map generated in the camera’s reference frame was translated to the robot’s reference frame using the adaptive transformation of the concept discussed in Section 4.1.8 under the vision system with a pan-tilt mechanism. Both maps were superimposed on one another using point-to-point coordinate correspondence to create a more useful SLAM-defect map. The fusion of the SLAM-based grid map and the defect occupancy map was performed using the overlay process discussed in [39].

### 5.5. Comparison with Existing Work

This section elaborates on the comparative analysis of the proposed algorithm with the existing drain inspection framework reported in the literature based on inspection tools and inspection algorithms. Table 6 states the accuracy of various inspection models and algorithms based on some similar classes.

Table 6 [3,4,5,42] used the CCTV as an inspection tool and applied different DL frameworks, namely Resnet-TL, four-layer custom CNN, five-layer custom CNN, and Modified ZF to automate the drain inspection. Here, Resnet-TL [3], and four [42] and five-layer CNN [5] are classifier frameworks that scored average precisions of 85%, 87.7%, and 78% (accuracy) for drain inspection tasks, respectively. Modified ZF [4] is an object detector framework and uses Faster RCNN as the detector head. The implementation scored an average precision of 83% across four different classes: root, crack, infiltration, and deposit.

In contrast with our implementation, the above schemes’ classification and detection scores are quite low; however, the implementations cannot be compared directly with our work since their datasets, CNN topology, and training parameters are different. Furthermore, the proposed framework utilizes IoRT-based remote real-time inspection in comparison with the above schemes, which utilizes offline inspection. This difference is reflected in the choice of the algorithm used.

Table 7 shows a comparative analysis of the current robotic platform with existing drain inspection platforms. Here, robots KURT, PIRAT, and MAKRO adopted a fixed morphology and were designed only for sewer pipe inspection with a small diameter. In contrast, Tarantula is a reconfigurable drain inspection robot. However, the robot has a high cost, has a complex mechanical design, and is heavyweight due to its many actuators. Moreover, its maneuverability is not stable in some drain segments [17]. On the other hand, our in-house developed robot Raptor is lighter and has a simplistic design approach. Additionally, its three modes of reconfiguration provide good maneuverability in varied terrain conditions. This design consideration is the core contribution of the proposed design with respect to the state-of-the-art.

## 6. Conclusions

An AI-enabled remote drain inspection and defect mapping framework was proposed using our in-house developed reconfigurable robot Raptor, the Faster RCNN object detection algorithm, and the IORT framework. The efficiency of the proposed system was tested through a robot maneuverability test, drain defect detection, and defect mapping accuracy. The reconfigurable robot was tested in three different drain environments in maneuverability tests: s2, V type, and flat terrain drains. The experimental results proved that the Raptor robot maneuverability was stable and that its defect mapping and localization are more precise using G mapping in the complex drain. Furthermore, the defect detection was tested using both offline collected defect images and real-time drain images collected by the Raptor robot. The experimental results show that the Faster RCNN Inception-ResNet-v2 variant has a good trade-off between detection accuracy and computation time compared with other variants. It obtained a 92.67% defect detection accuracy for offline defect detection experiments and an 89% defect detection accuracy for real-time Raptor-collected drain environment video streams. It took 119 ms to process one image on the local server. The overall experimental results proved that the proposed system is more suitable for automated defect detection task in drains and enhances the inspection service.

## Figures and Tables

**Table 1:** Technical specification of robot Raptor.

Description	Specification
Platform Weight	2.45 kg
Payload	Up to 1.6 kg
Dimensions	0.390 × 0.350 × 0.200 m
Environmental	3D-printed nylon for prototyping
Ground Clearance	0.098 m stowed, 0.150 m unstowed
Maximum Linear Velocity	0.22 m/s
Maximum Angular Velocity	0.85 rad/s
Maximum Gradient	20–25 degree
Maximum Side Gradient	18–20 degree
Traverse Terrain	Tested on short grassland, concrete, and road conditions

**Table 2:** Extracted feature layers.

Model Name	First Stage Feature Extractor	Second Stage Feature Extractor
ResNet50	block_1, block_2, block_3, block_4a	block_4
ResNet101	block_1, block_2, block_3, block_4a, block_4b	block_4
Inception-ResNet-v2	conv2d (1a, 2a, 2b, 3b, 4a), mixed_5b, mixed_6a, block_17, block_35	conv2d_7b, mixed_7a, block_8

**Table 3:** Statistical measures for defect detection.

Test	ResNet50				ResNet101				Inception-ResNet-v2			
	Prec.	Recall	$F_{1}$	Accuracy	Prec.	Recall	$F_{1}$	Accuracy	Prec.	Recall	$F_{1}$	Accuracy
Tree root intrusion	86.21	85.92	85.89	86.19	88.45	88.21	88.01	88.42	91.34	91.03	90.82	91.27
Plant intrusion	87.56	87.38	87.09	87.49	89.48	89.27	88.93	89.39	93.98	93.72	93.67	93.92
Crack	87.34	86.98	86.79	87.29	91.11	90.89	90.82	91.05	91.94	91.78	91.71	91.96
Pothole	86.71	86.49	86.37	86.69	90.87	90.65	90.58	90.79	92.43	92. 27	92.09	92.38
Bughole	85.97	85.63	85.59	85.95	89.33	89.02	88.94	89.29	93.82	93.61	93.55	93.73
Deposit	87.73	87.59	87.41	87.71	89.94	89.79	89.51	89.88	92.87	92.63	92.59	92.81

**Table 4:** Computationaltime analysis.

Model Name	Local Server
Faster RCNN ResNet50	95 ms
Faster RCNN ResNet101	300 ms
Faster RCNN Inception-ResNet-v2	119 ms

**Table 5:** Faster RCNN Inception-ResNet-v2 true positives and false positives.

			Day		Night			Day		Night	
Weather	Drain Type	Classes	TP	FP	TP	FP	Weather	TP	FP	TP	FP
Summer	Open	Treeroot	86	3	83	7	After Rainfall	82	5	79	7
		Plant	85	4	80	6		82	4	79	7
		Crack	86	3	83	6		83	4	81	8
		Pothole	87	4	84	5		83	5	81	5
		Bughole	83	5	80	8		79	4	78	6
		Deposit	84	5	80	8		79	4	78	5
	Semi-closed	Treeroot	85	4	81	6		83	5	80	7
		Plant	84	5	81	8		82	5	80	7
		Crack	87	3	83	6		84	4	82	6
		Pothole	82	6	80	8		80	5	79	6
		Bughole	83	4	80	6		81	5	79	7
		Deposit	83	4	81	5		81	4	80	7
	Closed	Treeroot	84	5	80	8		80	4	79	6
		Plant	83	6	80	7		81	6	80	7
		Crack	85	4	81	7		82	5	79	6
		Pothole	84	5	81	6		81	4	79	6
		Bughole	86	4	83	6		83	4	78	7
		Deposit	82	5	80	7		80	5	78	8

**Table 6:** Comparison analysis with existing object detection frameworks.

Case Studies	Inspection Type	Algorithm	Classes	Precision
Tennakoon et al. [3]	Offline CCTV	Resnet-TL	5	85.00
Kumar et al. [42]	Offline CCTV	4 layer CNN	3	87.7
Moradi et al. [5]	Offline CCTV	5 layer CNN	1	78.002
Cheng et al. [4]	Offline CCTV	Modified ZF	4	83.0
Proposed framework	Real-time with Raptor	Faster RCNN Inception-ResNet-v2 1	6	92.67

**Table 7:** Comparison with existing drain inspection robots.

	Tarantula [17]	MAKRO [16]	KURT [15]	PIRAT [12]	Raptor [43]
Morphology	Reconfigurable	Fixed-Shape	Fixed-Shape	Fixed-Shape	Reconfigurable
Weight (kg)	20	30	-	-	2.45
Dimension (m)	1.2	1.6	0.38 × 0.28 × 0.30	-	0.39 × 0.35 × 0.29
Speed (m/s)	-	0.3	0.20	0.35	0.22

## Data Availability

Data available on request due to restrictions.
